# Testing and assessment in psychology. A survey on Italian psychologists at the time of COVID-19 pandemic

**DOI:** 10.3389/fpsyg.2024.1345995

**Published:** 2024-04-19

**Authors:** Andrea Bobbio, Massimo Nucci, Roberta Daini, Filippo Aschieri, Daniela Traficante, Fiorenzo Laghi, Laura Parolin, Adriana Lis

**Affiliations:** ^1^Department of Philosophy, Sociology, Education, and Applied Psychology, University of Padova, Padua, Italy; ^2^Department of General Psychology, University of Padova, Padua, Italy; ^3^Department of Psychology, University of Milano-Bicocca, Milan, Italy; ^4^Department of Psychology, Catholic University of the Sacred Heart of Milan, Milan, Italy; ^5^Department of Developmental and Social Psychology, Sapienza University of Rome, Rome, Italy; ^6^Department of Developmental Psychology and Socialisation, University of Padova, Padua, Italy

**Keywords:** psychological testing, testing practice, testing use, online testing, European Federation of Psychologists Association (EFPA), COVID-19

## Introduction

Italian Universities offer undergraduate and graduate training on tests for students and are also the main providers of Continuous Education credit on test assessment for Italian Psychologists (Alessandri et al., 2021). With both students and professionals, teaching tests requires either to follow the most advanced scientific standards in the choice of which instruments to promote or wide spreading guideline for test use. A further complication in the field of teaching tests is finding a balance between two opposite positions: (1) giving theoretically driven knowledge and leaving that the specific tools will be learned by the psychologists at the beginning of their professional practice; (2) delivering a good knowledge and proficiency of most of the tools used at a given time in a specific community.

With the aim to offer a contribution to these issues, here we present the responses to a survey that involved the psychologists registered to the Italian Board of Psychologists. The survey aimed at providing: (a) the actual picture of psychological testing and assessment in the professional practice; (b) the attitudes toward the use of testing and the evaluation of the quality of the available instruments; (c) the quantity and quality of training received as regards psychological assessment at University, with a particular focus on Norm Referenced Tests (NRT).

We believe that the data presented here will be useful to improve teaching in psychology as well as professional training. Furthermore, the COVID-19 pandemic has accelerated the transition to tools to be administered remotely and the survey also covered this aspect.

## Norm-referenced tests

By NRT we refer to psychological assessment techniques which have been standardized in their administration, coding and interpretation, so that test-takers are evaluated in a similar way, irrespectively of where they live or who administers the test. NRT have been evaluated by researchers to assess the extent to which they are effective in measuring a particular psychological feature of respondents (e.g., cognitive functioning, personality traits, or disorders).

When used properly, NRT provide valuable individualized information on respondents on many potentially important aspects of their functioning, and help addressing specific challenges, clarifying diagnoses, and highlighting different strengths and difficulties (e.g., as regards interpersonal, intrapersonal, emotional, behavioral, and cognitive areas). NRT results support providing more effective prevention and intervention by planning tailored treatment for each individual. Also, in perspective, emphasis on NRT would prompt future generations of psychologists to accurately select tests based on their validity and reliability.

It is therefore of particular interest to know what psychologists think about tests, how they used them in general and in specific areas of psychology, and which challenges do they experience in NRT application. This data set includes information on Italian psychologists' attitudes toward NRT, and their online administration with a specific emphasis on the COVID-19 period. Data were collected within the framework of a survey promoted by the EFPA (European Federation of Psychologists Association) Board of Assessment.

## EFPA project on assessment and testing

In 2000, the EFPA promoted a survey on psychologists' attitudes toward various aspects of testing in six European countries (i.e., Belgium, Croatia, Slovenia, Spain, The Netherlands, and United Kingdom, Muñiz et al., 2001). The results showed that, in general, European psychologists had a positive attitude toward tests and testing. Their scores also indicated a desire for greater involvement of professional organizations in the regulation of tests along with more control on the qualification in their use. Concern over incorrect use of tests emerged as well. Moreover, results clearly indicated a request for further teaching, since University did not provide adequate training (Muñiz et al., 2001). Italy did not participate to this first investigation.

In 2009, the EFPA Standing Committee on Tests and Testing (EFPA-SCTT) reassessed European psychologists' opinions on tests (Evers et al., 2012). Seventeen European countries participated in the survey, and 11 countries previously not included participated to the study (i.e., Austria, Belgium, Croatia, Czech Republic, Germany, Greece, Lithuania, The Netherlands, Norway, Poland, Romania, Slovakia, Slovenia, Spain, Sweden, Turkey, and United Kingdom). Compared with the previous survey, in 2009 psychologists were more satisfied with information received about test quality, had less concern over quality standards in the use of test materials, but were more preoccupied about illegal copying of test manuals and materials. The new sample showed high appreciation of tests in all countries, but among countries the survey found differences with regards incorrect test use and need for regulations on tests and testing. Moreover, a new dimension was taken into account concerning Internet testing with a low score and high differences between countries (Evers et al., 2012). Italy did not participate also in this second wave investigation.

In 2012 the EFPA Board of Assessment issued a third survey on opinions about tests involving countries worldwide, and comparing results with the point of view of European's psychologists collected in 2009. The following new groups of respondents were included: Brazil, Bulgaria, China, Denmark, Hungary, Indonesia, Israel, Italy, Latvia, Lebanon, New Zealand, and Nigeria. The study was published in 2017 (Evers et al., 2017). Italy participated with a sample of 5,482 participants (80% female).

In 2019 the EFPA Board of Assessment decided to repeat the same survey with the questionnaire used in the 2009 and 2012 administration. In Italy the Assessment Group of the Italian Association of Psychology (AIP) promoted the participation of Italian psychologists belonging to the Italian Board of Psychologists. The EFPA questionnaire was inserted in a wider investigation dealing with (a) the actual picture of psychological testing and assessment in the professional practice; (b) the attitudes toward the use of testing and the evaluation of the quality of the available instruments; (c) the quantity and quality of training received as regards psychological assessment at University. At the we are writing this article, the overall results have not been disseminated yet. However, a comparison between Croatian and Italian psychologists' attitudes is available (Lis et al., 2022).

## COVID-19 pandemic

Starting from the beginning of 2020, the world found itself collapsing under the effects of the SARS-CoV-2 pandemic. The subsequent countermeasures to avoid contagious diseases included restrictions mainly aimed at decreasing social contacts and maintaining distance between people. In many countries the peak of the pandemic was managed by a strict lockdown, with little or no access to the hospitals except for acute illness.

All of this induced the need for online assessment. This in turn led to significant changes in clinical practice. Psychologists had to modify their settings to assess patients' mental status and cognitive abilities, moving from in-person to remote assessment by using video calling platforms and other resources. Related to this, our survey included a new part of the online assessment, mandatory during lockdown periods.

## Method

### Participants

A total of 2,412 Italian psychologists participated in the current study, a percentage of 2% of the 120,601 psychologists registered in the National Order of Psychologists^1^ in 2020. However, 922 people (38.2%) did not supply any answer and were deleted from the dataset. The final number of non-empty questionnaires gathered is 1,490.

Males were 229 (15.4% of the whole sample) and females were 1,252 (84%; nine missing values, 6%); mean age was equal to 45.41 years (*sd* = 11.84). Geographical area of living was recoded in three groups, as follows: Northern Italy, *n* = 953 (64%), Central Italy, *n* = 313 (21%) and Southern Italy, *n* = 224 (15%). As regards the level of education obtained after the Master's degree in Psychology, *n* = 447 (30%) owned a 2-year post-graduate degree, *n* = 983 (66%) a 4-year specialization degree enabling the exercise of psychotherapy, and *n* = 89 (6%) a Ph.D (multiple answers were allowed). To sum up, most respondents were self-employed (*n* = 1,039, 69.7%), worked in the Clinical psychology field (*n* = 1,000, 67%), declared more than 15 years of seniority as professional psychologist (*n* = 591, 39.6%), and more than 15 years of seniority as a member of the CNOP (*n* = 647, 43%; for details, see Table 1). While the representativeness of the sample was not formally assessed, our data seem to match the features of psychologists working in Italy, with a majority of middle-aged woman working as self-employed professionals and residing in the northern part of Italy.

**Table 1:** Descriptive statistics of items presented in Section 1 of the questionnaire and of selected items belonging to Section 2 and 3.

Number of participants	1,490					
Gender	M = 229 (15.4%)					
	*F* = 1,252 (84%)					
	Missing values = 9 (0.6%)					
Age	M = 45.42 (*SD* = 11.84)					
Geographic area	Northern Italy = 955 (64.1%)					
	Central Italy = 296 (19.9%)					
	Southern Italy = 255 (15.1%)					
	Missing values = 14 (0.9%)					
Level of education beyond the Master's Degree (the respondents could select more than one answer/category)	Post lauream Master's Degree = 459 (30.8%)					
	Post lauream Specialization Degree = 984 (66%)					
	Ph.D. = 96 (6.4%)					
Job contract	Employed in the public sector = 178 (11.9%)					
	Employed in the private sector = 156 (10.5%)					
	Self-employed = 1,039 (69.7%)					
	Atypical employment = 44 (3%)					
	Others = 64 (4.3%)					
	Missing values = 9 (0.6%)					
Field of specialization	Clinical = 1,000 (67.1%)					
	Neuropsychology = 77 (5.2%)					
	Work, marketing, and organization = 100 (6.7%)					
	Social and community = 63 (4.2%)					
	Development and education = 123 (8.3%)					
	Basic research (mainly academics) = 21 (1.2%)					
	Other = 88 (5.9%)					
	Missing values = 18 (1.2%)					
Number of years of registration in the National Board of Psychologists	<1 year = 51 (3.4%)					
	1–2 years = 88 (5.9%)					
	3–5 years = 187 (12.6%)					
	6–10 years = 235 (15.8%)					
	10–15 years = 275 (18.5%)					
	More than 15 years = 647 (43.4%)					
	Missing values = 7 (0.5%)					
**Answers to selected items**						
*The European Federation of Psychologists Associations (EFPA) should establish a European system to accredit the certification of test users*	1. Totally disagree = 116 (7.8%)					
	2. = 143 (9.6%)					
	3. = 298 (20%)					
	4. = 586 (39.3%)					
	5. Totally agree = 327 (21.9%)					
	Missing values = 20 (1.3%)					
*Do you use tests in your profession?*	Yes = 1,066 (71.5%)					
	No = 424 (28.5%)					
*Did you already use online tests before COVID-19?*	Yes = 195 (13.1%)					
	No = 660 (44.3%)					
	Missing values = 635 (42.6%)					
*In the current historical moment (COVID-19), how many times have you been in contact with your customers?*	1. Never	2. Occasionally	3. Rarely	4. Often	5. Very often	Missing values
*Telephone*	18 (1.2%)	86 (5.8%)	247 (16.6%)	328 (22%)	119 (8%)	692 (46.4%)
*E-mail*	146 (9.8%)	168 (11.3%)	250 (16.8%)	158 (10.6%)	51 (3.4%)	717 (48.1%)
*Chat*	179 (12%)	129 (8.7%)	213 (14.3%)	183 (12.3%)	57 (3.8%)	729 (48.9%)
*Video conference/online meeting*	55 (3.7%)	48 (3.2%)	116 (7.8%)	306 (20.5%)	271 (18.2%)	694 (46.6%)
*In person*	37 (2.5%)	77 (5.2%)	132 (8.9%)	322 (21.6%)	214 (14.4%)	708 (47.5%)
*Other*	92 (6.2%)	8 (0.5%)	16 (1.1%)	12 (0.8%)	11 (0.7%)	1,351 (90.7%)
*During the current historical moment (COVID-19), how many times have you administered online tests?*	546 (36.6%)	119 (8%)	81 (5.4%)	52 (3.5%)	16 (1.1%)	676 (45.4%)

### Measures

The questionnaire included three sections. *Section 1*. Ten questions asked respondents information on their personal and professional background (V2-V11; e.g., gender, age, geographical area, level of education, job contract, field of specialization, and number of years of registration in the National Board of Psychologists). *Section 2*. This part included 40 items. Thirty-two items belonged to the EFPA Questionnaire on Test Attitudes of Psychologists (EQTAP), i.e., items Q1_1 to Q1_24, plus the eight options of item Q1_25 (i.e., items Q1_25_1 to Q1_25_8, see Evers et al., 2012, p. 318, Appendix A). Moreover, eight newly developed questions on the same issues (i.e., Q2_1 to Q2_8). All items, were scored on a 5-point Likert-type scale ranging from 1 = completely disagree/very rarely to 5 = completely agree/very frequently. The items assessed several topics, such as the quality of training received in Psychology, legislation and deontology issues, and administration procedures of psychological testing (e.g., “The training received in Psychology Bachelor's Degree courses is sufficient for the correct use of most tests” and “Legislation is needed to control the more serious abuse of testing” from EQTAP; “People to which a test has been administered have the right to receive an explanation of results” from new items). *Section 3*. This section comprised 47 items, of which and adaptation of n. 26 of the EQTAP (i.e., items Q1_26_1 to Q1_26_3, requiring text format answers, e.g., “Write the name of the three tests you use most frequently in carrying out your profession—Test 1”; see Evers et al., 2012, p. 318, Appendix A). Items from Q3 to Q10 dealt with the respondents' experience with tests (e.g., Q3: “Do you use tests in your work/profession?” answer: yes/no), online testing administration (e.g., Q5_1: “The use of online tests, remotely, is completely similar to the use of in-person tests” answer: yes/no), and professional experience and testing with particular reference to the lockdown due to COVID-19 (e.g., Q5_11: “The current historical moment (COVID-19) can provide the opportunity to learn new digital processes such as online consultation or online testing”). When the response scale was not dichotomous or required a percentage, items were scored on a 5-point Likert-type scale, ranging from 1 = completely disagree/very rarely to 5 = completely agree/very frequently.

### Procedure

The survey was administered online through the Qualtrics platform. All members of the CNOP were invited to participate via e-mail on a voluntary basis. The survey was open for answers for 3 months, starting from January 2021 to May 2021. A reminder was sent 4 weeks after the first e-mail.

Overall, of the 1,490 people who provided valid questionnaires, 1,449 answered to at least 75% of items of Section 1 and 2, and 796 to at least 75% of items of all the three Sections. Table 1 summarizes key information on the gathered data along with descriptive statistics concerning selected items. Items displayed in Table 1 were selected based on their salience for readers interested in the demographic of participants and in their general approach to online testing.

## Strengths and limitations

The dataset provides a starting point for a better understanding of current views on testing according to a group of Italian professional psychologists. Moreover, it would make it possible to compare them with previous surveys and with those collected in other nations, and to plan strategies to improve the use of testing instruments and their teaching in academia.

As regards limitations, the self-selected convenience sample of respondent was suboptimal if compared with the actual number of psychologists enrolled to the CNOP. Respondents were not entirely representative of the population in terms of geographic area of residence, field of specialization, and number of years of registration in the National Board of Psychologists.

## Possible research paths

Based on the presented dataset, scholars can conduct numerous analyses concerning a broad range of research questions on a cross-cultural level, with regard to, for instance, age, gender, level of education, country of origin, academic training for the use of tests, area in which practitioners use psychological tests. In addition, scientists may apply these data to identify predictors of psychological testing use.

## Dataset description

The dataset contains the answers of 1,490 Italian psychologists to a questionnaire about the use of assessment tools in their professional practice, which includes a part concerning online testing administration during COVID-19 pandemic.

## Data availability statement

The datasets presented in this study can be found in the Open Science Framework (OSF) repository. The names of the repository/repositories and accession number(s) can be found at: https://osf.io/2p3gh/?view_only=b2baaae724874f7e94fe3825fc207fbf. The deposit contains two files: Database.xlsx, containing the raw data; Codebook.xlsx, containing item codes, item descriptions, and response scale/options.

## Ethics statement

The studies involving humans were approved by Ethical Committee for Research in Psychology (CERPS) of the Catholic University of the Sacred Heart of Milan (code 62-23). The studies were conducted in accordance with the local legislation and institutional requirements. The participants provided their written informed consent to participate in this study.

## Author contributions

AB: Data curation, Formal analysis, Investigation, Methodology, Writing—original draft, Writing—review & editing. MN: Data curation, Formal analysis, Investigation, Methodology, Writing—original draft, Writing—review & editing. RD: Investigation, Writing—original draft, Writing—review & editing. FA: Conceptualization, Investigation, Methodology, Writing—review & editing. DT: Conceptualization, Investigation, Methodology, Writing—review & editing. FL: Conceptualization, Investigation, Methodology, Writing—review & editing. LP: Investigation, Methodology, Resources, Writing—review & editing. AL: Conceptualization, Investigation, Supervision, Writing—original draft, Writing—review & editing.

## Footnotes

^1^The Law n. 56 of the 18th of February 1989, and the Ministerial Decree n. 240 of the 13th of January 1992, altogether established the organization of the profession of psychologist in Italy. The CNOP (National Council of the Order of Psychologists) was founded in 1992, and all professionals who want to practice the profession in Italy need to become registered psychologists after passing a state exam.
